# Inhibitory Effect of IL-1β on HBV and HDV Replication and HBs Antigen-Dependent Modulation of Its Secretion by Macrophages

**DOI:** 10.3390/v14010065

**Published:** 2021-12-30

**Authors:** Marion Delphin, Suzanne Faure-Dupuy, Nathalie Isorce, Michel Rivoire, Anna Salvetti, David Durantel, Julie Lucifora

**Affiliations:** 1 CIRI–Centre International de Recherche en Infectiologie, Univ Lyon, Université Claude Bernard Lyon 1, Inserm, U1111, CNRS, UMR5308, ENS Lyon, 69007 Lyon, France; marion.delphin@inserm.fr (M.D.); anna.salvetti@inserm.fr (A.S.); david.durantel@inserm.fr (D.D.); 2 INSERM, U1052, Cancer Research Center of Lyon (CRCL), University of Lyon (UCBL1), CNRS UMR_5286, Centre Léon Bérard, 69008 Lyon, France; suzanne.fauredupuy@gmail.com (S.F.-D.); nathalie.isorce@unil.ch (N.I.); 3 INSERM, U1032, Centre Léon Bérard, 69008 Lyon, France; michel.rivoire@lyon.unicancer.fr

**Keywords:** hepatitis D virus, hepatitis B virus, interleukine-1 beta, macrophages, NF-κB, hepatocytes, antiviral activity

## Abstract

Co-infection with the hepatitis B virus and hepatitis delta virus (HDV) leads to the most aggressive form of viral hepatitis. Using in vitro infection models, we confirmed that IL-1β, a crucial innate immune molecule for pathogen control, was very potent against HBV from different genotypes. Additionally, we demonstrated for the first time a strong and rapid antiviral effect induced by very low doses of IL-1β against HDV. In parallel, using co-culture assays, we demonstrated that monocytes exposed to HBV, and in particular to HBsAg, during differentiation into pro-inflammatory macrophages secreted less IL-1β. Altogether, our data emphasize the importance of developing combined antiviral strategies that would, for instance, reduce the secretion of HBsAg and stimulate the immune system to produce endogenous IL-1β efficient against both HBV and HDV.

## 1. Introduction

Chronic hepatitis B virus (HBV) infection affects more than 250 million people worldwide and is responsible for severe liver diseases [1]. Co-infection with the hepatitis delta virus (HDV) leads to the most aggressive form of viral hepatitis [2]. The HBsAg, secreted by infected cells, induces deregulation of both innate and adaptive immune cells [3,4]. While the control of HBV viremia can be reached in chronic hepatitis B patients treated with nucleos(t)ides analogues (NA) that target the reverse transcription of HBV, a functional cure is rarely achieved and HDV replication is not affected by NA treatment in co-infected patients [1,2]. Functional cure of HBV is defined by HBsAg < 0.05, HBV DNA not detectable and normal ALT in the absence of therapy, whereas functional cure for HDV is defined by HDV RNA not detectable with normal ALT in the absence of therapy. Of note, functional cure of HDV is harder to achieve than functional cure of HBV (as shown by the HIDIT-I and HIDIT-II clinical trials [5,6]). New antivirals currently being investigated for HBV/HDV include, for instance, molecules targeting the assembly function of core (HBc) or inhibiting HBsAg synthesis/secretion, siRNAs targeting HBV RNAs, and innate immune modulators [1]. This is of importance, since the decrease of HBsAg could potentially restore anti-HBV immune responses as reviewed here [3].

## 2. Materials and Methods

### 2.1. Primary Cells Purification and Cells Culture

HepaRG cells were cultured and differentiated as previously described [7]. HepAD38 and HepG2 cells were cultured as previously described [8] and treated with tetracycline (Sigma-Aldrich, Saint-Louis, MO, USA; final concentration 1 µg/mL) for 20 days to fully inhibit HBeAg and HBV Dane particles secretion. Monocytes from healthy donors were isolated and cultivated as previously described [9]. Monocytes were seeded into regular for 24-well plates or 6.3 mm diameters PET inserts for 24-well plates, with pores of 0.45 uM diameter (Dominique Dutscher, Bruxelles, Belgium; 2515127) when co-cultured. Monocytes were exposed to 50 ng/mL of GM-CSF (R&D System, Minneapolis, MN, USA) during 6 days for differentiation into M1-MDM. M1-MDM were activated by stimulation with 10 ng/mL of LPS (Invivogen, San Diego, CA, USA) for 3 h. Cells were washed three times with PBS and cultured in fresh medium for another 3 h before a last medium exchange. Supernatants and cells were collected 24 h post-stimulation (i.e., 18 h accumulation). Monocytes were co-cultured with hepatocytes (HepG2 or HepAD38 cells) during their differentiation and stimulation step (i.e., insert removed after 3 h LPS stimulation).

### 2.2. Viral Inoculation

Differentiated HepaRG (dHepaRG) or PHH were cultured and infected with HBV (100 vge/cell) and/or HDV (10 vge/cell) (PEG-precipitated) as previously described [10,11]. Blood monocytes were exposed to HBV inocula (or control medium) prepared by concentrating supernatant from HepAD38 [12] by ultrafiltration. All viral inocula were tested for the absence of endotoxin (Lonza, Bale, Switzerland) and characterized by analyses of the fractions from a 5.6–56% iodixanol gradient and analyzed by ELISA, dot blot with HBV DIG-labelled probe [13], and Western blot (HBc, DAKO, B0586). This allowed us to rule out the presence of non-enveloped nucleocapsids (data not shown) that may activate immune cells [14]. Blood monocytes were exposed to at least three different batches of HBV (full inoculum concentrated by ultrafiltration) at a multiplicity of infection of 1000 vge/cell. Viral inocula from HBV genotype C and E were similarly prepared from the supernatant of a newly developed, stably transformed HepG2 cell lines. Briefly, cell lines were obtained by transfection of a linearized pcDNA3Neo-HBV plasmid containing 1.35 genome unit of a consensus sequence of HBV genotype C or E (obtained from HBV database: https://hbvdb.lyon.inserm.fr accessed on 23 December 2021, sequences are available upon request) and a double-round selection under G418 (500 ng/mL) by colony cell cloning (very low density seeding in large flasks).

### 2.3. Cytokine and Drugs

Unless indicated otherwise, cells were treated once with the following molecules without renewal of the media: IFN-α (Roferon, Roche, Bâle, Switzerland; final concentration used to treat the cells: 500 UI/mL), hIL-1β (JM4128-10, MBL, Woburn, MA, USA; final concentration used to treat the cells: 100 pg/mL), Anakinra (Kineret, Sobi, Stockholm, Sweden; final concentration used to treat the cells: 0.04 mg/mL), TPCA-1 (Sigma-Aldrich, Saint-Louis, MO, USA; final concentration used to treat the cells: 1 µM), and RG7834 (AI-Biopharma, Montpellier, France; final concentration used to treat the cells: 1 µM).

### 2.4. SiRNA Treatment

HepAD38 cells were transfected once a week with a negative control pool of four siRNAs that we named siRNA Ctrl (D-001810) or siRNA targeting all the HBV transcripts (SAHIP-000001, seq: CGACCUUGAGGUAUACUUCUU), both purchased from Dharmacon Inc. (Lafayette, CO, USA) and using Lipofectamine RNAiMAX reagent (Life Technologies, Gaithersburg, MA, USA; 13778150) according to the manufacturer’s instructions. Three weeks were required to obtain full inhibition of HBV secreted parameters using this technique.

### 2.5. Quantification of Secreted Proteins by ELISA

HBeAg and HBsAg secretion were quantified using chemiluminescence immunoassay kit (Autobio, Zhengzhou Henan, China), and cytokines’ secretion was analyzed using Duoset ELISA (R&D system, Minneapolis, MN, USA), both following the manufacturer’s instructions.

### 2.6. RNA Extraction and RT-qPCR

Total RNA from hepatocytes were extracted with RNAzol according to the manufacturer’s instructions. cDNAs were synthetized using the Maxima RT (Thermo Scientific™, Life Technologies, Villebon-sur-Yvette, France) according to the manufacturer’s instructions. qPCR analyses were performed using Luna (Invitrogen, Carlsbad, CA, USA). mRNA expression was assessed by comparative cycle threshold (Ct) method (2 − ΔCt); RPLP0 was used as housekeeping genes. Primers sequences are the following for detection of all HBV RNAs (Forward: GGAGGGATACATAGAGGTTCCTTGA; Reverse: GTTGCCCGTTTGTCCTCTAATTC), all HDV RNAs (Forward: CGGGCCGGCTACTCTTCT; Reverse: AAGGAAGGCCCTCGAGAACA), RPLP0 (Forward: CACCATTGAAATCCTGAGTGATGT; Reverse: TGACCAGCCCAAAGGAGAAG), MxA (Forward: GGTGGTCCCCAGTAATGTGG; Reverse: CGTCAAGATTCCGATGGTCCT), OAS (Forward: AGGTGGTAAAGGGTGGCTCC; Reverse: ACAACCAGGTCAGCGTCAGAT), IL-6 (Forward: TCGAGCCCACCGGGAACGAA; Reverse: GCAACTGGACCGAAGGCGCT), and A20 (Forward: TCCTCAGGCTTTGTATTTGAGC; Reverse: TCTCCCGTATCTTCACAGCTT).

### 2.7. Statistical Analysis

Results are presented as means ± standard deviations and analyzed for statistical significance using the Mann–Whitney *U* test with Prism software. Significant *p*-values are indicated by asterisks in the figure legends. *: *p* < 0.05; **: *p* < 0.01; ***: *p* < 0.001; ****: *p* < 0.0001.

## 3. Results and Conclusions

**Figure 1:**
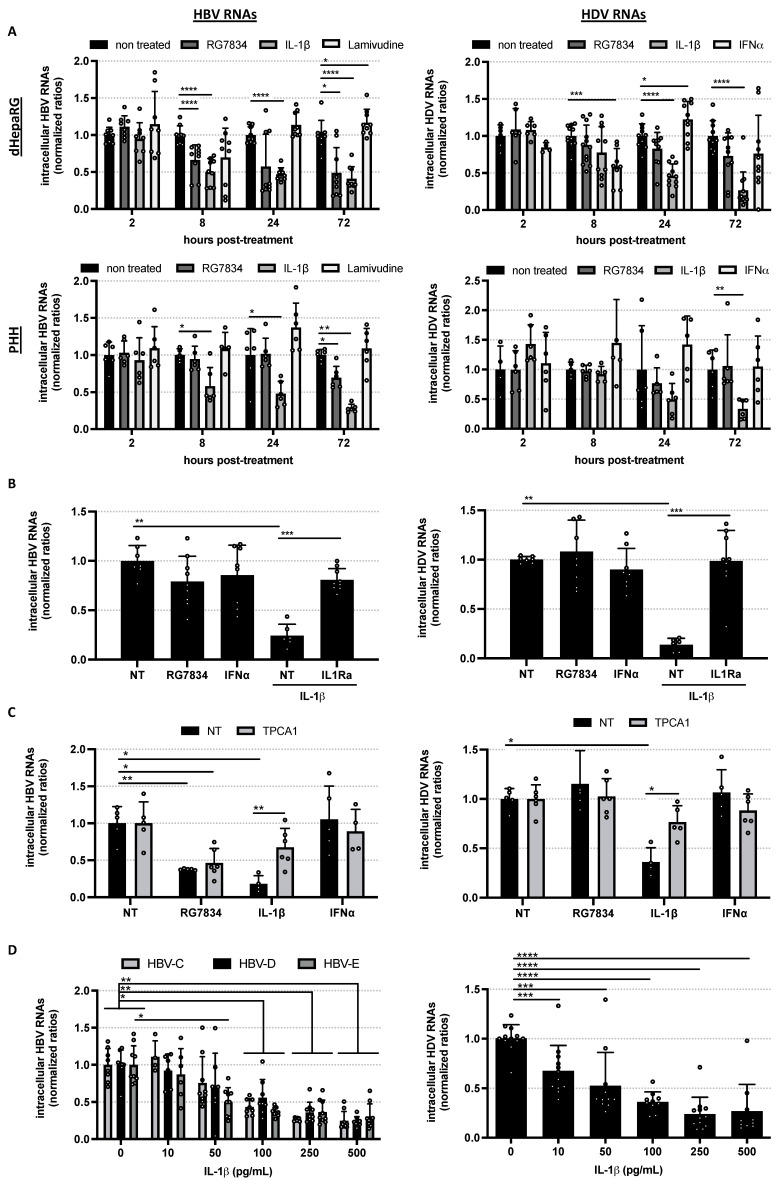
IL-1β-mediated activation of IL-1R triggers NF-κB activation and decreases the levels of intracellular HBV RNAs from different genotypes as well as HDV RNAs. (**A**) dHepaRG and PHH infected with HBV-D alone or co-infected with HDV were treated 7 or 4 days later, respectively, with the indicated molecules for the indicated time (2 h, 8 h, 24 h, or 72 h). (**B**,**C**) HBV-D/HDV co-infected dHepaRG were treated 7 days later with (**B**) IL-1Ra or (**C**) TPCA1 24 h prior and during the 3 days of treatment with IL-1β. (**D**) dHepaRG infected with HBV-C to -E or HDV were treated 7 days later with increasing doses of IL-1β for 3 days. (**A**–**D**) Cells were harvested, total RNAs were extracted, and levels of viral RNAs were quantified by RT-qPCR. Results are the means ± SD of at least two independent experiments each performed with three biological replicates represented as dots on each graph. Significant p-values are indicated by asterisks in the figure legends. *: *p* < 0.05; **: *p* < 0.01; ***: *p* < 0.001; ****: *p* < 0.0001.

Using in vitro infection models, we and others previously demonstrated that IL-1β, a crucial innate immune molecule for pathogen control, is the most potent cytokine acting against HBV (genotype D) with an IC50 of 50 pg/mL [15,16]. Here, we showed that levels of intracellular HBV RNAs are decreased very early after treatment with IL-1β in differentiated HepaRG (dHepaRG) or primary human hepatocytes (PHH) (Figure 1A, left panels) with a kinetics similar to the one observed with the HBV RNAs destabilizer RG7834 known to target the polyA tails of HBV RNAs [17]. Importantly, we also observed a rapid reduction of HDV RNA levels up to 50% upon a 24h stimulation of dHepaRG cells or PHH with IL-1β (Figure 1A, right panels). Notably, no antiviral effect was observed at this time in cells treated with NA (Lamivudine) or IFN-α (Figure 1A). IFN-α is another innate immune cytokine, which is a backbone component of the treatment of chronic HDV patients [2], but requires repeated and longer treatments to be effective against HBV and HDV in vitro [10]. As expected, in coinfected hepatocytes exposed to IL-1β, prototypic NF-κB genes (IL-6 and A20) were induced (Figure S1A). Prototypic interferon-stimulated genes (ISGs) (OAS1 and Mx1) were not induced upon exposure to IL-1β, contrary to what is usually observed upon exposure to IFN-α (Figures S1A and S1B). Using an IL-1R antagonist (IL1Ra) [18] or an inhibitor of IKKβ (TPCA-1) [19], we demonstrated that the antiviral effect of IL-1β on both HBV and HDV is mediated by the stimulation of the IL-1R (Figure 1B) and the subsequent activation of the NFκB pathway (Figure 1C). As several genotypes have been described for HBV, each having specific geographic distribution and physiopathologic features [7], we also tested the antiviral effect of IL-1β on two other HBV genotypes (E and C) and showed similar antiviral efficacy with a dose-dependent decrease in HBV-C, HBV-D, HBV-E, and HDV RNAs levels and an IC50 estimated at 50–100 pg/mL (Figure 1D). Altogether, our data demonstrate a stronger (i.e., induced by very low doses) and faster antiviral effect of IL-1β against both HBV and HDV than current molecules used as standard of care, paving the way to therapeutic approaches aiming at inducing endogenous IL-1β production.

However, our previous studies demonstrated that IL-1β secretion by macrophages/myeloid cells was impaired in the presence of HBV-D [9]. Here, we extended this observation to HBV-C, as IL-1β and IL-6 secretions by pro-inflammatory monocyte-derived-macrophages (M1-MDMs) were reduced upon LPS stimulation in the presence of both HBV-D and HBV-C, compared to cells not exposed to the virus during differentiation (Figure S2). Considering that macrophages are the main producers of IL-1β in the human body [18,20], we aimed to identify the HBV protein(s) responsible for macrophages modulations in order to be able to set up efficient antiviral strategies inducing and/or restoring endogenous IL-1β production.

**Figure 2:**
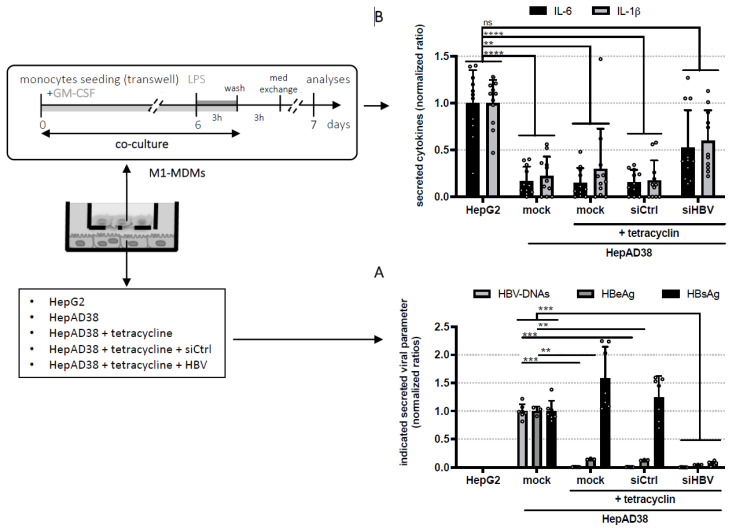
HBV surface proteins are responsible for the impairment of IL-1β and IL-6 secretion by macrophages. HepG2 or HepAD38 were treated with tetracyclin and/or transfected with siRNA as described in the Materials and Methods section. (**A**) Supernatants were harvested to quantify secreted HBV DNA, HBeAg, and HBsAg by qPCR and ELISA. Results are expressed as ratios normalized to the condition without “HepAD38 mock”. (**B**) Cells were then co-cultured with monocytes during their differentiation as indicated in the scheme. Seven days post-seeding, supernatants of M1-MDMs were harvested and analyzed for content in IL-1β and IL-6 by ELISA. Results are expressed as ratios normalized to the condition without HBV, i.e., “HepG2”. Results are the mean ± SD of at least three independent experiments each performed with three biological replicates represented as dots on each graps. Significant p-values are indicated by asterisks in the figure legends. *: *p* < 0.05; **: *p* < 0.01; ***: *p* < 0.001; ****: *p* < 0.0001.

To date, HBeAg, HBsAg, or the HBV capsid protein (HBc) have been suggested to contribute to the inhibition of macrophages responses [20], with most of the studies using recombinant proteins or correlations with the HBV antigens status of patients to draw their conclusions. Here, to be in a more relevant setting and constant exposure to viral products, we further explored this question by co-cultivating monocytes (during their differentiation into M1-MDMs) and HepAD38 cells with a transwell-based system that we previously described [9]. As expected, HBV Dane particles and HBeAg secretion in HepAD38 cells could be abrogated by treatment with tetracyclin without affecting HBsAg release [8] (Figure 2A). Alternatively, the secretion of all HBV particles and antigens could be abrogated by transfecting these cells with siRNAs targeting all HBV RNAs (Figure 2A). M1-MDMs were differentiated in co-culture with HepAD38, previously treated or not with tetracyclin and/or transfected with control siRNA or siRNA against HBV, or with HepG2 (the parental cell line that does not express HBV) before stimulation with LPS. We confirmed [9] that the presence of HBV particles and antigens lead to a 80% decrease of IL-1β and IL-6 secretion of stimulated M1-MDMs (Figure 2B). Interestingly, the same impairment was observed when M1-MDMs were differentiated only in the presence of HBsAg (HepAD38 cells treated with tetracyclin) (Figure 2B). HBsAg-induced reduction of IL-1β and IL-6 was partially reversed when M1-MDMs were co-cultured with HepAD38 cells transfected with siRNA against HBV RNAs (Figure 2B).

Of note, even though unlikely, we cannot exclude the possible involvement of HBx in HBV-induced macrophages modulations since tet-treated HepAD38 can produce the HBx mRNAs that could eventually be transmitted to macrophages through exosomes [21].

Altogether, our data confirmed the immuno-modulatory effect of HBsAg on macrophages and emphasize the importance of developing a combined antiviral strategy that would both reduce the secretion of HBsAg and stimulate the immune system to produce antiviral pro-inflammatory cytokines efficient against both HBV and HDV.

## Data Availability

The data presented in this manuscript are available through the corresponding author upon reasonable request.

## Supplementary Materials

The following are available online at https://www.mdpi.com/article/10.3390/v14010065/s1, Figure S1: IL-1β treatment leads to increase expressions of genes from the NFkB pathway but not from the IFN pathway and Figure S2: Decreases of IL-1β and IL-6 secretion when M1-MDM were differentiated in the presence of HBV from genotype D and C.

## References

[1] Bartoli A., Gabrielli F., Tassi A., Cursaro C., Pinelli A., Andreone P. (2021). Treatments for HBV: A Glimpse into the Future. Viruses.

[2] Rizzetto M. (2016). The Adventure of Delta. Liver Int..

[3] Tout I., Loureiro D., Mansouri A., Soumelis V., Boyer N., Asselah T. (2020). Hepatitis B Surface Antigen Seroclearance: Immune Mechanisms, Clinical Impact, Importance for Drug Development. J. Hepatol..

[4] Vaillant A. (2021). HBsAg, Subviral Particles, and Their Clearance in Establishing a Functional Cure of Chronic Hepatitis B Virus Infection. ACS Infect Dis..

[5] Wranke A., Hardtke S., Heidrich B., Dalekos G., Yalçin K., Tabak F., Gürel S., Çakaloğlu Y., Akarca U.S., Lammert F. (2020). Ten-Year Follow-up of a Randomized Controlled Clinical Trial in Chronic Hepatitis Delta. J. Viral. Hepat..

[6] Bremer B., Anastasiou O.E., Hardtke S., Caruntu F.A., Curescu M.G., Yalcin K., Akarca U.S., Gürel S., Zeuzem S., Erhardt A. (2021). Residual Low HDV Viraemia Is Associated HDV RNA Relapse after PEG-IFNa-Based Antiviral Treatment of Hepatitis Delta: Results from the HIDIT-II Study. Liver Int..

[7] Kramvis A. (2014). Genotypes and Genetic Variability of Hepatitis B Virus. INT.

[8] Ladner S.K., Otto M.J., Barker C.S., Zaifert K., Wang G.H., Guo J.T., Seeger C., King R.W. (1997). Inducible Expression of Human Hepatitis B Virus (HBV) in Stably Transfected Hepatoblastoma Cells: A Novel System for Screening Potential Inhibitors of HBV Replication. Antimicrob. Agents Chemother..

[9] Faure-Dupuy S., Delphin M., Aillot L., Dimier L., Lebossé F., Fresquet J., Parent R., Sebastian Matter M., Rivoire M., Bendriss-Vermare N. (2019). Hepatitis B Virus-Induced Modulation of Liver Macrophage Function Promotes Hepatocyte Infection. J. Hepatol..

[10] Alfaiate D., Lucifora J., Abeywickrama-Samarakoon N., Michelet M., Testoni B., Cortay J.-C., Sureau C., Zoulim F., Dény P., Durantel D. (2016). HDV RNA Replication Is Associated with HBV Repression and Interferon-Stimulated Genes Induction in Super-Infected Hepatocytes. Antivir. Res..

[11] Luangsay S., Gruffaz M., Isorce N., Testoni B., Michelet M., Faure-Dupuy S., Maadadi S., Ait-Goughoulte M., Parent R., Rivoire M. (2015). Early Inhibition of Hepatocyte Innate Responses by Hepatitis B Virus. J. Hepatol..

[12] Iwamoto M., Watashi K., Tsukuda S., Aly H.H., Fukasawa M., Fujimoto A., Suzuki R., Aizaki H., Ito T., Koiwai O. (2014). Evaluation and Identification of Hepatitis B Virus Entry Inhibitors Using HepG2 Cells Overexpressing a Membrane Transporter NTCP. Biochem. Biophys. Res. Commun..

[13] Lucifora J., Salvetti A., Marniquet X., Mailly L., Testoni B., Fusil F., Inchauspé A., Michelet M., Michel M.-L., Levrero M. (2017). Detection of the Hepatitis B Virus (HBV) Covalently-Closed-Circular DNA (CccDNA) in Mice Transduced with a Recombinant AAV-HBV Vector. Antiviral Res..

[14] Cooper A., Tal G., Lider O., Shaul Y. (2005). Cytokine Induction by the Hepatitis B Virus Capsid in Macrophages Is Facilitated by Membrane Heparan Sulfate and Involves TLR2. J. Immunol..

[15] Isorce N., Testoni B., Locatelli M., Fresquet J., Rivoire M., Luangsay S., Zoulim F., Durantel D. (2016). Antiviral Activity of Various Interferons and Pro-Inflammatory Cytokines in Non-Transformed Cultured Hepatocytes Infected with Hepatitis B Virus. Antiviral Res..

[16] Watashi K., Liang G., Iwamoto M., Marusawa H., Uchida N., Daito T., Kitamura K., Muramatsu M., Ohashi H., Kiyohara T. (2013). Interleukin-1 and Tumor Necrosis Factor-α Trigger Restriction of Hepatitis B Virus Infection via a Cytidine Deaminase Activation-Induced Cytidine Deaminase (AID). J. Biol. Chem..

[17] Han X., Zhou C., Jiang M., Wang Y., Wang J., Cheng Z., Wang M., Liu Y., Liang C., Wang J. (2018). Discovery of RG7834: The First-in-Class Selective and Orally Available Small Molecule Hepatitis B Virus Expression Inhibitor with Novel Mechanism of Action. J. Med. Chem..

[18] Boraschi D., Italiani P., Weil S., Martin M.U. (2018). The Family of the Interleukin-1 Receptors. Immunol. Rev..

[19] Nan J., Du Y., Chen X., Bai Q., Wang Y., Zhang X., Zhu N., Zhang J., Hou J., Wang Q. (2014). TPCA-1 Is a Direct Dual Inhibitor of STAT3 and NF-ΚB and Regresses Mutant EGFR-Associated Human Non-Small Cell Lung Cancers. Mol. Cancer Ther..

[20] Faure-Dupuy S., Durantel D., Lucifora J. (2018). Liver Macrophages: Friend or Foe during Hepatitis B Infection?. Liver Int..

[21] Kapoor N.R., Chadha R., Kumar S., Choedon T., Reddy V.S., Kumar V. (2017). The HBx Gene of Hepatitis B Virus Can Influence Hepatic Microenvironment via Exosomes by Transferring Its MRNA and Protein. Virus Res..

