# Supplementary material for: Inhibitory Effect of IL-1β on HBV and HDV Replication and HBs Antigen-Dependent Modulation of Its Secretion by Macrophages
Source: Viruses. 2021 Dec 30;14(1):65. doi: 10.3390/v14010065 (PMC8781515; doi:10.3390/v14010065)
Supplement: Supplementary file 1 [file viruses-14-00065-s001.zip › viruses-1494943-supplementary.pdf]

viruses

MDPI

Supplementary Material

Inhibitory effect of IL-1β on HBV and HDV replication and HBs antigen-dependent modulation of its secretion by macrophages

Marion Delphin, Suzanne Faure-Dupuy, Nathalie Isorce, Michel Rivoire, Anna Salvetti, David Durantel and Julie Lucifora

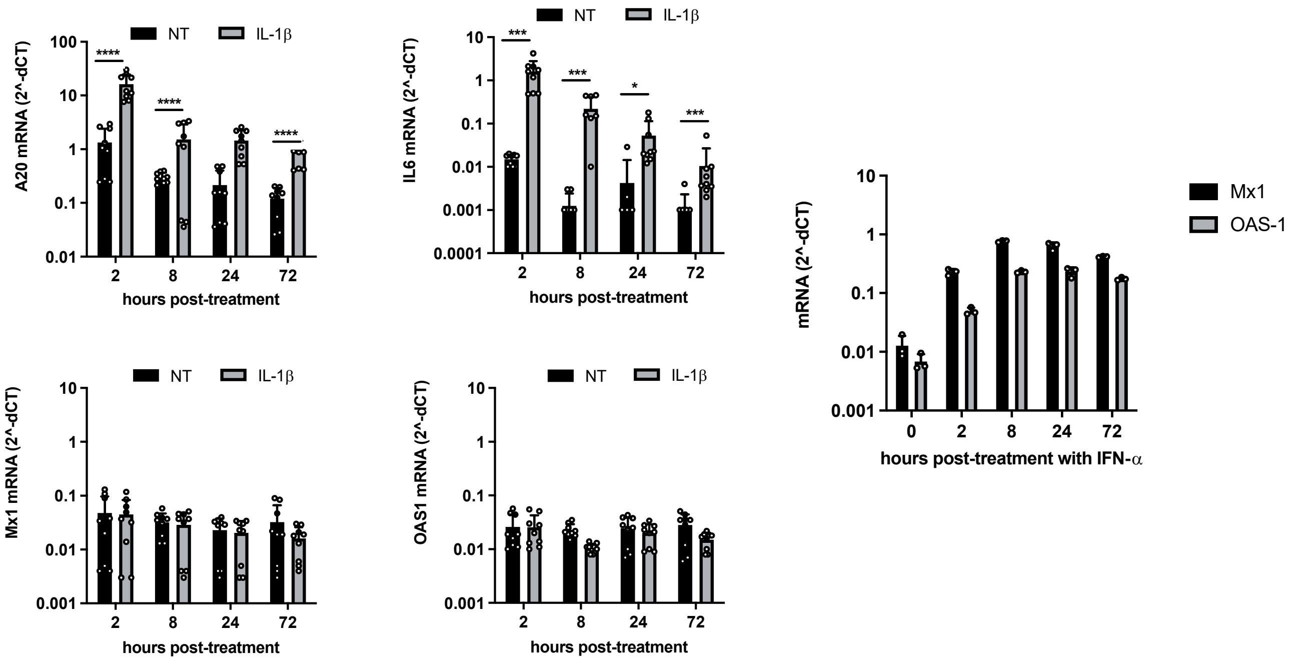

(a) (b)

Figure S1. IL-1β treatment leads to increase expressions of genes from the NFkB pathway but not from the IFN pathway. (**A**) dHepaRG infected with HBV-D were treated or not 7 days later with IL-1β for the indicated time. Cells were harvested, total RNA extracted and levels of the indicated mRNA were quantified by RT-qPCR. At least two independent experiments with three biological replicates are shown. (**B**) dHepaRG cells were treated with IFN-*α* (500 IU/mL) for the indicated time. Cells were harvested, total RNA extracted and levels of the indicated mRNA were quantified by RT-qPCR. One experiment with three biological replicates is shown.

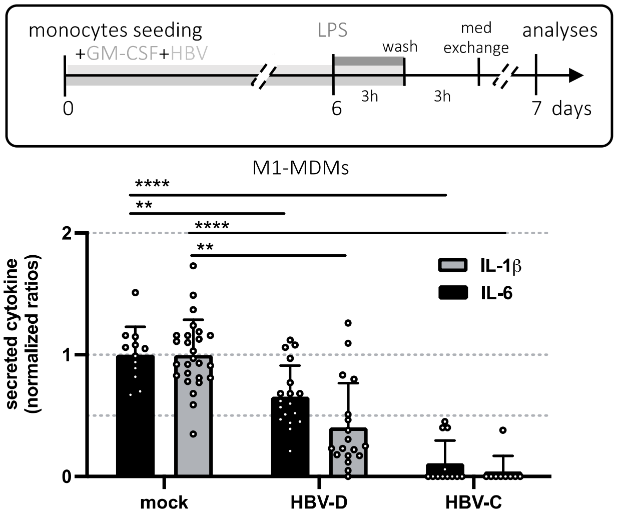

Figure S2. Decreases of IL-1β and IL-6 secretion when M1-MDM were differentiated in the presence of HBV from genotype D and C. M1-MDMs were incubated with HBV-C or HBV-D during differentiation and stimulation with LPS, as indicated. Four independent experiments (i.e., four different donors) with four biological replicates are shown.
